# Adherence of Brazilian Minors to the 24-Hour Movement Guidelines after In-Person School Return

**DOI:** 10.3390/ijerph21070930

**Published:** 2024-07-17

**Authors:** Natália Molleri, Saint Clair Gomes Junior, Daniele Marano, Andrea Zin

**Affiliations:** Instituto Nacional de Saúde da Mulher, da Criança e do Adolescente Fernandes Figueira/FIOCRUZ, Rio de Janeiro 22250-020, Brazil; saint.junior@fiocruz.br (S.C.G.J.); danielemarano@yahoo.com.br (D.M.); andrea.zin@fiocruz.br (A.Z.)

**Keywords:** fidelity to guidelines, COVID-19 pandemic, public health, physical exercise, screen time, sleep, infant, preschool, child, adolescent

## Abstract

The levels of adequacy of movement behaviors after returning to in-person school activities following the COVID-19 pandemic are not yet well understood. This study aimed to assess the adherence of Brazilian minors to the recommendations of the 24-Hour Movement Guidelines (moderate to vigorous physical activity, recreational screen time, and sleep duration), as well as overall adherence to these guidelines, after the relaxation of COVID-19 social isolation measures and the resumption of in-person schooling. A cross-sectional study was conducted with parents or guardians (39 ± 7.8) of minors aged up to 18 years of age (3.8 ± 2.5). A total of 172 individuals responded to the questionnaire. Data were compared with those obtained in the Survey of the Adequacy of Brazilian Children and Adolescents to the 24-Hour Movement Guidelines before and during the COVID-19 Pandemic. There was an 18.6 percentage decrease (*p*-value < 0.001) in overall adherence to the 24-Hour Movement Guidelines when comparing the periods before the COVID-19 pandemic (March 2020) and after the return to in-person schooling (March 2021). The largest percentage drop in adherence between these periods was observed for moderate to vigorous physical activity (27.4%; *p*-value < 0.001) and the lowest for sleep (10.5%; *p*-value < 0.001). Adherence to the 24-Hour Movement Guidelines did not return to pre-pandemic levels with the resumption of in-person school activities.

## 1. Introduction

Breaks on weekends or during school holidays negatively influence the practice of physical activity and increase the use of screens and electronic devices in school-age children and adolescents [1,2,3,4]. The COVID-19 pandemic has severely disrupted the movement behaviors of children and adolescents due to social distancing measures, with greater impact on populations subjected to more stringent restrictions [5,6,7,8]. The health crisis has altered domestic and school routines, affecting the socio-emotional status of children and adolescents with systematic reviews reporting increased anxiety, depression, and hyperactivity [9,10,11,12]. The pandemic has also exacerbated global rates of food insecurity [13,14,15]. In Brazil, thousands of children were affected by the lack of meals provided by their schools [14,16,17].

Several studies, utilizing the 24-Hour Movement Guidelines [18,19], have demonstrated that children and adolescents exhibited reduced physical activity as well as increased recreational screen time and sleep duration during the pandemic compared to the period before [20,21,22,23,24,25]. Research conducted prior to the pandemic suggested that the inadequacy of these behaviors affected the motor development and physical well-being of children, contributing to increased adiposity, exacerbated cardiometabolic conditions, and potentially resulting in emotional and behavioral alterations [26,27,28,29].

It is worth noting that there have been only a limited number of studies assessing the adherence to the 24-Hour Movement Guidelines (moderate to vigorous physical activity (MVPA), recreational screen time and sleep duration) following the return to in-person schooling [30,31]. According to Pfledderer et al. [30], attending school in-person has a positive influence on meeting the overall recommendations of the three components of the 24-Hour Movement Guidelines and MVPA and screen time individually. Another study highlighted the benefits of returning to school for meeting MVPA guidelines among 4th to 6th graders (*n* = 300), while nearly 80% of older children and adolescents (*n* = 500), did not meet MVPA recommendations [31]. The limited exploration of this topic underscores the significance of this study. The components of the 24-Hour Movement Guidelines reflect good health-related habits, and understanding adherence to these guidelines can provide opportunities to enhance overall health and well-being.

In Brazil, existing socioeconomic inequalities had a more profound impact on the course of the COVID-19 epidemic than factors such as age, health conditions, and other disease risk factors. This disparity imposed a disproportionate burden on state and municipal administrations leading to high socioeconomic vulnerability [32]. Social distancing measures aimed at curbing the spread of COVID-19 resulted in approximately 39 million children and adolescents enrolled in the public basic education system to be kept away from schools [33]. A survey conducted by the National Institute of Educational Studies and Research Anísio Teixeira (INEP) revealed that, on average, face-to-face activities were suspended for 279 days during the 2020 school year in both public and private schools [34]. In contrast, countries like France and Portugal experienced significantly shorter suspensions of in-person classes, with 43 and 67 days, respectively [35].

Given the return to in-person school activities, our hypothesis was that a structured day (represented by a school day) would restore minors’ adherence to the 24-Hour Movement Guidelines to pre-pandemic levels. The purpose of this study was to assess the adherence of Brazilian minors to the recommendations of the 24-Hour Movement Guidelines (moderate to vigorous physical activity, recreational screen time, sleep duration, and overall adherence to these three components) following the relaxation of isolation measures and the resumption of in-person schooling.

## 2. Materials and Methods

### 2.1. Study Design, Configuration, and Participants

**Figure 1:**
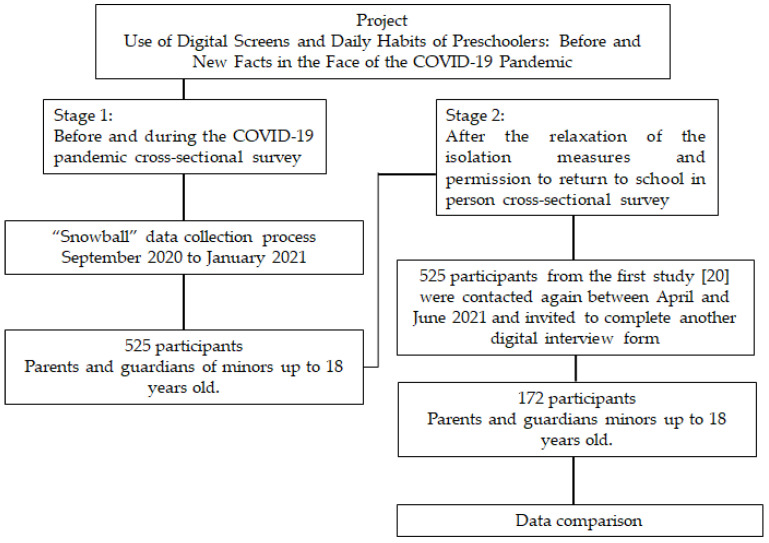
Flow diagram of participant recruitment.

This study represents the second stage of data collection for the project “Use of Digital Screens and Daily Habits of Preschoolers: Before and New Facts in the Face of the COVID-19 Pandemic”, conducted between April and June 2021, following the return to in-person school activities after one year since the COVID-19 pandemic was declared. The project utilized online data collection, divided into two distinct stages: before and during the COVID-19 pandemic, and after the return to in-person schooling. Parents and guardians of children and teenagers up to 18 years old participated. The first stage of data collection occurred between September 2020 and January 2021, during the period of social isolation measures due to COVID-19. A convenience sample was gathered using a “snowball” data collection process [36], where participants agreed to complete a questionnaire electronically distributed via social networks. Data from 525 participants were collected, analyzed, and previously published [20]. These 525 participants from the initial study [20] were contacted again and invited to complete another digital interview form (Figure 1).

All subjects gave their informed consent for inclusion before they answered the questionnaire. This research received approval from the Institutional Review Board of the Instituto Nacional de Saúde da Mulher, da Criança e do Adolescente Fernandes Figueira/FIOCRUZ (approval number 4.277.985) and adhered to the *Checklist for Reporting Results of Internet E-surveys* (CHERRIES) [37] and *The Strengthening the Reporting of Observational Studies in Epidemiology* (STROBE) guidelines [38].

### 2.2. Instruments and Data Collection

For comparison purposes, the present study utilized an electronic data collection form developed on a digital platform (Google Forms). The form consisted of 30 questions addressing the sociodemographic characteristics of families, MVPA, recreational screen time, and sleep duration. This allowed data comparison with first stage reported by Molleri et al. [20]. The estimated time for parents and guardians to complete the form was approximately 20 min.

The sociodemographic characteristics of parents or guardians and minors were classified into the following categories: region of the country (South; Southeast; Midwest; North and Northeast); age; gender (male; female); ethnicity (white; brown/black); higher education (no; yes); participation in distance learning (no; yes); employment status at the time of form completion and change since March 2020 (employed/unemployed); remote work (no; yes); current average family income (below 4 minimum wages; between 4 and 10 minimum wages and above 10 minimum wages, considering that the Brazilian minimum wage in 2021 was BRL 1100.00); and household income loss as of March 2020 (no; yes).

The average duration of MVPA, recreational screen time, sleep, and the combined adherence to all three parameters (overall adherence) before and after the resumption of in-person school activities were classified as either adequate or inadequate based on the 24-Hour Movement Guidelines [18,19]. The responses were categorized as follows: average MVPA time—less than 30 min per day, 30 min per day, 1 h per day, 1.5 h per day, and more than 1.5 h per day; average daily recreational screen time (television, mobile, computer, laptop, or tablet)—none, less than 1 h, 1 h, 2 h, 3–4 h, 5–7 h, and more than 8 h; and average sleep duration (including naps)—15 or more hours per day, 13–14 h per day, 11–12 h per day, 8–10 h per day, and less than 8 h per day.

### 2.3. Outcomes

The outcomes were defined based on the guidelines by Tremblay et al. [18,19] regarding adherence to the 24-Hour Movement Guidelines. The specific recommendations for each component are outlined below:

1. MVPA: Infants who are not yet walking should engage in 30 min of activity throughout the day in a prone position (belly down) or playing away from screens; children aged 1–2 should have at least 180 min of physical activity per day; and children aged 3 and older should aim for 60 min of MVPA daily [18,19].
2. Screen time: Children under 2 should not be exposed to digital screens; children aged 2–4 should have no more than one hour of screen time per day; and children aged 5 and above should limit screen time to two hours per day [18,19].
3. Sleep duration (including naps): Infants aged 0–3 months should sleep for 14–17 h per day; infants aged 4–11 months should aim for 12–16 h per day; toddlers aged 1–2 should have 11–14 h of sleep; children aged 3–5 should aim for 10–13 h; children aged 6–13 should sleep for 9–11 h per day; and children aged 14–17 should aim for 8–10 h of sleep per day [18,19].
4. Overall adherence: participants’ simultaneous adherence to the recommendations for MVPA, recreational screen time, and sleep duration [18,19].

### 2.4. Data Analysis

Data were automatically exported from Google Forms to an Excel spreadsheet. The data from this study were then compared with the data from the initial cross-sectional study of the project, as published by Molleri et al. [20]. The comparative data were analyzed using the JASP 0.16.1 statistical software package. Categorical variables were presented with their respective frequencies (both absolute and relative), while numerical variables were summarized using the mean and standard deviation. McNemar’s test was conducted to assess the differences in sociodemographic variables and the adherence to MVPA, recreational screen time, sleep duration, and overall adherence before and after the Brazilian government’s relaxation of isolation measures and the resumption of in-person school activities. In all the analyses, a significance level of *p* < 0.05 was applied.

## 3. Results

A total of 172 parents or guardians completed the questionnaire, and their responses were compared with those from the previous study. All parents or guardians had attained higher education; 160 resided in the Southeastern region (93%) with a mean age of 39 years. The average age of the minors was 3.8 years, with 147 (85.5%) of them being under 6 years old. Participants indicated that 66 (38.4%) of the minors had transitioned back to exclusively in-person schooling. Additional details regarding the sociodemographic characteristics of the participants and their adherence to the 24-Hour Movement Guidelines before the COVID-19 pandemic and following the resumption of in-person school activities can be found in Table 1.

The sample size was insufficient to demonstrate significant differences for variables such as in-person education, male gender of the person in-charge, black or brown skin color, unemployment, face-to-face work, and family income below four minimum wages (Table 1).

The most substantial percentage drop in adherence between the periods before COVID-19 and after the resumption of in-person schooling was observed for MVPA (27.4%; *p*-value < 0.001), while the lowest decline was noted for sleep (10.5%; *p*-value < 0.001). Additional details on adherence to the 24-Hour Movement Guidelines are provided in Table 2.

There was an 18.6% decrease (*p*-value < 0.001) in overall adherence to the 24-Hour Movement Guidelines (including MVPA, recreational screen time, and sleep duration) between the periods before and after the return to in-person schooling (Table 2). It is important to note that even during the best period, which was before the pandemic, the overall adherence was only 24.40% (Table 2).

## 4. Discussion

While we anticipated that adherence to the 24-Hour Movement Guidelines would resemble pre-pandemic levels, as indicated by other studies [30,31], we discovered that they were actually even lower based on the reports of parents and guardians.

The lack of centralized coordination in managing the health crisis within academic settings and the delayed vaccination rollout for children and adolescents may have influenced our findings [39]. Furthermore, underlying these issues was the politicization of the health crisis. The management of the health crisis in Brazil has been characterized by the absence of a crisis committee established by the federal government to define rules and protocols for pandemic control [40]. This void led to individual states and municipalities implementing isolated measures with varying degrees of stringency in terms of isolation and mobility restrictions [41]. The lack of coordination between the federal government, states, and municipalities [42] has likely contributed to the uncertainties stemming from insufficient or conflicting information, which has been a significant factor in the reluctance of parents/guardians and education professionals to resume face-to-face activities. Recognizing the importance of resuming in-person teaching and learning activities across all levels of national basic education, the Brazilian government [43] issued official recommendations. However, despite these guidelines, only an average of 10% of Brazilian schools returned to in-person activities during the 2020 school year, with varying rates at different levels: federal (2% return), municipal (3% return), state (15% return), and private (30% return) [44]. This trend continued throughout 2021, with schools remaining closed for in-person activities for 51% of the school year [34]. This differed from the situation in other countries like Argentina, Chile, Italy, and Spain, where 100% of classroom education had already resumed [34,35].

Moreover, there was a delay in initiating COVID-19 vaccination for children and adolescents in comparison to developed countries. Most developed nations implemented priority vaccination plans and commenced vaccination earlier than low- and middle-income countries like Brazil [45]. Despite the pandemic dynamics being influenced by the population’s socioeconomic and demographic factors, the vaccination rollout in Brazil was marked by controversies, debates, and political exploitation of the health crisis [39,46,47]. The vaccination campaign began on 17 January 2021, in the state of São Paulo, focusing on frontline healthcare workers and other high-risk groups such as the elderly, immunocompromised individuals, and those with chronic illnesses [48]. Vaccination for adolescents against COVID-19 in Brazil only commenced on 15 September 2021, followed by the start of vaccination for children on 14 January 2022 [49,50].

Therefore, merely returning to school was not sufficient to restore adherence to the 24-Hour Movement Guidelines after the pandemic. Among the survey respondents, we observed an approximate 19% decline in overall adherence, with MVPA dropping by around 27%, screen time increasing by about 20%, and sleep duration decreasing by approximately 11%. Consequently, the “Structured Days Hypothesis” proposed by Brazendale et al. [3] is refuted, as a structured day (represented here by a school day) did not shield children from obesogenic behaviors. It is important to note that the data evaluated pertain to a theoretically less vulnerable population; thus, it is likely that the general population faces an even more critical risk of obesity.

It is important to highlight that the combination of low physical activity, sedentary behavior (such as excessive use of digital screens), and inadequate sleep has been linked to significant negative health outcomes. These include cardiovascular issues (such as metabolic syndrome, high blood pressure, and risk factors for cardiovascular diseases), increased adiposity (body fat, weight, and waist circumference), changes in cognitive development (including language development, attention, and executive functions), and mental disorders (such as low self-esteem, anxiety, and depression) [51,52,53,54]. The literature strongly supports the importance of the interaction of all movement behaviors throughout the day, rather than focusing on a single movement behavior in isolation [26,27,51,55].

According to the World Health Organization (WHO), levels of physical inactivity tend to increase as countries develop economically [56]. In some nations, inactivity rates can reach as high as 70%, driven by changes in transportation, increased use of technology, cultural values, and urbanization. The WHO highlights that a sedentary lifestyle can lead to higher healthcare costs, environmental impacts, hindered economic development, and diminished community well-being and quality of life. To mitigate these issues, the WHO has proposed a physical activity policy initiative aimed at achieving the 2030 Sustainable Development Goals: reducing sedentary behavior by 10% by 2025 and by 15% by 2030. In line with this initiative, the Brazilian Ministry of Health published a document in 2021 focusing on promoting health through physical activity among the Brazilian population. The *Physical Activity Guide for the Brazilian Population* [57] offers the first set of recommendations and information from the Ministry of Health on physical activity, encouraging an active lifestyle to enhance health and improve quality of life. The guide also provides strategies for reducing sedentary behavior, particularly in relation to screen time, although it does not address sleep duration as a component of movement behaviors.

The primary limitation of our study is the recruitment method, which employed a “snowball” sampling strategy [36]. Initially, the investigators distributed the survey to their contacts, who were then encouraged to share it with their own social networks. This approach has several limitations, including a lack of control over sample composition, lack of representativeness and precision, susceptibility to sampling bias, and potential homogeneity within the sample, as participants may share certain characteristics with the researchers. Consequently, this limits the generalizability of the results to the Brazilian middle class, which historically has had greater access to health and education services. However, considering the social determinants affecting the Brazilian population, the strength of this study lies in its ability to speculate on the adherence rates to the 24-Hour Movement Guidelines among the most vulnerable segments of the population. This is particularly significant given that low adherence was identified even within our predominantly middle-class sample.

## 5. Conclusions

The relaxation of social isolation measures and the return to in-person school activities were not sufficient for improving adherence to MVPA, recreational screen time, sleep duration, and overall adherence to the 24-Hour Movement Guidelines. This study did not identify positive effects of structured routines on movement behaviors. It is crucial to develop more effective strategies to enhance adherence to the 24-Hour Movement Guidelines, particularly in low-and middle-income countries like Brazil, not only to return to but also surpass pre-pandemic levels of overall adherence. This is essential for improving health-related habits within the population.

## Figures and Tables

**Table 1:** Sociodemographic variables and overall adherence before the COVID-19 pandemic and after the Brazilian government’s relaxation of the isolation measures and the resumption of in-person school activities.

Variables		Overall Adherence before the COVID-19 Pandemic		Overall Adherence after Relaxation for School Return		
		Yes	No	Yes	No	
	*n* (%)	*n* (%)	*n* (%)	*n* (%)	*n* (%)	*p*-Value ^2^
Minors	172 (100)	42 (24.4)	130 (75.6)	10 (5.8)	162 (94.2)	<0.001
Mean age (standard deviation)	3.8 (2.5)					
Gender						
Male	87 (50.6)	21 (24.1)	66 (75.9)	3 (3.4)	84 (96.6)	<0.001
Female	85 (49.4)	21 (24.7)	64 (75.3)	7 (8.2)	78 (91.8)	0.003
Education						
In-person	66 (38.4)	12 (18.2)	54 (81.8)	5 (7.6)	61 (92.4)	0.092
Distance or hybrid	106 (61.6)	30 (28.3)	76 (71.7)	5 (4.7)	101 (95.3)	<0.001
Parents or Guardians	172 (100)					
Region of the country						
Southeast	160 (93.0)					
Other	12 (0.7)					
Mean age (standard deviation)	39 (7.8)					
Gender						
Male	16 (9.3)	2 (12.5)	14 (87.5)	0 (0.0)	16 (100.0)	0.500
Female	156 (90.7)	40 (25.6)	116 (74.4)	10 (6.4)	146 (93.6)	<0.001
Ethnicity						
White	118 (68.6)	31(26.3)	87 (73.7)	6 (5.1)	112 (94.9)	<0.001
Black or brown	54 (31.4)	11(20.4)	43 (79.6)	4 (7.4)	50 (92.6)	0.118
Employment status						
Unemployed	16 (9.3)	5 (31.2)	11 (68.8)	2 (12.5)	14 (87.5)	0.375
Employee	156 (90.7)	37 (23.7)	119 (76.3)	8 (5.1)	148 (94.9)	<0.001
Remote work (responsible)						
No	49 (28.5)	6 (12.2)	43 (87.8)	4 (8.2)	45 (91.8)	0.727
Yes	117 (68.0)	36 (30.8)	81 (69.2)	6 (5.1)	111 (94.9)	<0.001
Missing	6 (3.5)	-	-	-	-	
Family income ^1^						
<4 mw	25 (14.5)	5 (20.0)	20 (80.0)	3 (12.0)	22 (88.0)	0.687
Between 4 and 10 mw	63 (36.6)	14 (22.2)	49 (77.8)	2 (3.2)	61 (96.8)	<0.001
≥10 mw	84 (48.9)	23 (27.4)	61 (72.6)	5 (5.9)	79 (94.1)	<0.001
Loss of family income						
No	89 (51.7)	20 (22.5)	69 (77.5)	7 (7.9)	82 (92.1)	0.004
Yes	83 (48.3)	22 (26.5)	61 (73.5)	3 (3.6)	80 (96.4)	<0.001

^1^ Brazil’s minimum wage in 2021: BRL 1100.00; ^2^ McNemar’s signed-rank test comparing the distribution of each category, before X after; mw: minimum wage.

**Table 2:** Adherence to the 24-Hour Movement Guidelines (moderate to vigorous physical activity, recreational screen time, and sleep duration) before the COVID-19 pandemic and after the Brazilian government’s relaxation of the isolation measures and the resumption of in-person school activities.

		Adherence before the COVID-19 Pandemic	Adherence after Relaxation for School Return	*p*-Value ^2^
		*n* = 172		
	Variables	*n* (%)	*n* (%)	
MVPA ^1^		120 (69.8)	73 (42.4)	<0.001
Screen time	Television	111 (64.5)	54 (31.4)	<0.001
	Cell phone	138 (80.2)	99 (57.6)	<0.001
	Tablet, computer or notebook	153 (89.0)	119 (69.2)	<0.001
Sleep duration		107 (62.2)	89 (51.7)	0.018
Overall adherence		42 (24.4)	10 (5.8)	<0.001

^1^ Moderate to vigorous physical activity. ^2^ McNemar’s signed-rank test.

## Data Availability

The raw data supporting the conclusions of this article will be made available by the authors on request.
